# Our Country

**Published:** 1876-05

**Authors:** 


### OUR COUNTRY.

THE mechanics and farmers are the pillars of any nation of enduring greatness. Of the two classes, the farmer is the most important. Taking out now and then the brightest intellect and the best heart for doctors and clergymen, it would be a grand thing for our country, if every young man, at the completion of his twenty-first year, was required to be a practical farmer or a finished mechanic, and this to continue for fifty years, and thus allow all politicians, lawyers, gamblers, drunkards, dandies, go-betweens, gentlemen-loafers, bachelors, and all such dispensable characters, to die off. A plan like this would give a stability to our nation beyond all that whig or democrat ever dreamed of.

We have a territory large enough, north of the Missouri and west of the Mississippi rivers, large enough to give a good-sized farm to every competent agriculturist that can be prepared for it, in the next half century, while the mechanics will build their houses, erect their mills, dig their canals, and stretch out their railroads; a territory as large in extent as that of the twenty-four States, east of the Mississippi river—vast plains of territory teeming with vegetation, and swarming with animal life—plains which pasture twenty millions of buffalo, and provide shelter for fifty millions more of wild animals of every description.

On these magnificent plains, a thousand miles broad, "without a single abrupt mountain, timbered space, desert, or lake, running smoothly out to the navigable waters of the St. Lawrence, the Mississippi, the Missouri rivers, and the Texan coast," with scarce a rock, or stone, or tree within the circuit of many miles on these great plains—now swarming with millions of buffalo, the Yankee of another century will make his mark, the mark of the church and the school-house, the plantation and the city.

But without wood or yet discovered coal, how would the teeming millions of a score of States live? The great God is always wise and always good. His benevolence precedes the need of man, his child, by untold ages. There is wood enough growing under all these plains to last for uncounted generations, to be had anywhere for the digging; for the dry atmosphere stunts the vegetation above ground, and all the strength goes to the roots, which spread out in all directions as large as a man's arm.

By a late-discovered document, it is ascertained that a census of the Chinese empire was taken in 1852, and that it amounted to three hundred and ninety-six millions of souls. We have an uninhabited country beyond the Mississippi, equally large and quite as capable of sustaining an equal population, and it will be peopled by our own race, speaking our own language, and maintaining, we trust, our own religion, the religion of the Bible. What work! how boundless the field for the clergyman, the schoolmaster, the physician, to give them morals, learning, and health!

These things are worth the matured thoughts of all the good, of the philanthropist, the sage, and the Christian. May the church, the school-house, the manufactory, and the farm, all do their meet and just share in founding well that great nation yet to come.

